# Proceedings of the Pathological Society of Philadelphia

**Published:** 1858-07

**Authors:** 


ART. IV.—*Proceedings of the Pathological Society of Philadelphia.* Reported by the SECRETARY.

WEDNESDAY EVENING, April 14th, 1858.

The President, DR. GROSS, in the chair.

*Abscess of the Ovary Communicating with the Sigmoid Flexure of the Colon.*—DR. STILLÉ exhibited a specimen of ovarian abscess, for which he was indebted to Dr. Bell. The abscess had evidently discharged into the colon; an opening was found on the side of the ovary, and a corresponding opening in the sigmoid flexure. The communication was fistulous, and appeared to be of long standing. The peritoneum showed signs of inflammation; the liver was rather larger than normal, and fatty; there was no tubercular disease of the mesenteric glands; the bronchial glands were enlarged, and apparently filled with a deposit. No history of the case could be procured.

DR. GROSS spoke of the comparative rarity of ovarian abscesses. He also mentioned that a physician of this city had reported a case of an abscess of the ovary containing a gallon of pus.

DR. HARRIS had seen a case of supposed ovarian dropsy tapped, in which, doubtlessly, an abscess of the ovary had existed. About two gallons of an extremely offensive pus were drawn off.

DR. DARRACH believed that ovarian cysts might contain an extremely offensive fluid resembling pus, yet it would be hardly right to call such formations abscesses. He cited two cases to prove his assertion. In one, the ovarian cyst filled the whole abdomen, and was mistaken for ascites, on account of the existence of a tympanitic sound above the line of effusion, resulting from the elimination of gases within the cyst; the fluid evacuated was extremely offensive. In the second case, the cyst also contained a purulent fluid. In both, the walls were lined by a dense, flaky layer of lymph.

DR. AGNEW accounted for the fact that the abscess communicated with the intestine at the sigmoid flexure, by supposing that the latter must have been displaced by fæces, which it often is in women. He had frequently, in the dissecting room, met with ovarian cysts, but never with abscesses.

DR. STILLÉ would not positively affirm that the specimen before the Society was an abscess. The walls were rough, and the appearances were those of an abscess; yet it might have been a cyst.

*Isolated Fibrous Tumor from the Mammary Gland.*—DR. PACKARD, in presenting this specimen, made the following remarks:—This tumor was given to me by my friend Dr. Turnbull, who removed it about two weeks ago from the breast of an unmarried lady, aged twenty-one. It had been growing a little over three years, was freely movable, and projected somewhat above the level of the skin at the upper part of the gland. The patient thought she could trace it to an injury. Her menstrual functions were very slightly, if at all deranged. When the mass was removed, a small pedicle was observed connecting it with the adjoining gland-tissue.

Under the microscope there were seen a vast number of free nuclei, some oval, and others very much elongated and even curved. Many well-characterized fibro-plastic cells were also present, and very delicate fibrous tissue in large quantity. Acetic acid had little or no effect on these structures.

The diagnosis, before the removal of this tumor, was, that it belonged to the class described by Velpeau as *adenoid.* Sir Astley Cooper's *chronic mammary* tumors, and Cruveilhier's *fibrous,* are identical with these; Nélaton follows Lebert, whom he quotes freely, in calling them *partial hypertrophies* of the gland. Lebert's account of their structure is very full: he says they present no other elements than those of the gland, and they may affect either the fibrous tissue or the proper gland-structure. In the former case, he states, they have a fibro-gelatinous appearance, and are the only kind of fibrous tumor to which the breast is liable. He also mentions that they have a cellular envelope, and are easily enucleated, but that their independence of the surrounding tissues is only apparent; a pedicle being generally traceable. The existence of this pedicle is denied by Velpeau.

The cells of the present growth had, to my eye, very much the appearance of ordinary fibro-plastic cells, while the conversion of nuclei into fibres was traceable with admirable distinctness.

*Meningitis of the Convex Surface of the Brain; Tubercular Deposit in the Lungs.*—DR. MORTON exhibited a brain removed from T. D——, aged thirty, who was admitted into the Pennsylvania Hospital on the 3d of April, 1858. He was dull and stupid when he came into the hospital, but stated that he had been sick for two weeks with constant headache, and had bled much from the nose. He presented no marked symptoms, except dullness of mind, trifling cough, and a slight amount of paralysis of the lower limbs. He was, however, very weak. His condition remained the same, with the exception of his mind becoming duller, until the 9th, when he had a violent chill, followed by a convulsion which lasted about an hour, and left him perfectly comatose. His pulse now rose; his urine passed involuntarily from him; difficulty in swallowing became a marked symptom, and he continued to sink until the 11th, when he died. The treatment consisted in cold water to the head, blisters and dry cups to the neck, purging, small doses of mercury, varied with stimulants and supporting remedies.

Post-mortem examination.—The lungs were somewhat congested, and contained patches of infiltrated tubercle. In the heart were large fibrinous clots, the largest occupied the left auricle and ventricle. The stomach and intestines were congested; the cœcum was especially so; the liver, kidneys, and spleen were healthy; the brain showed evidences of recent inflammation; large patches of lymph were deposited on its superior and its anterior surfaces. The same patches were found in the fissure of Sylvius. Under the dura mater, in the course of the longitudinal sinus, at the upper and posterior portion of the brain, some degenerate lymph and pus were met with. The dura mater itself was, here and there, along the course of the longitudinal sinus, firmly attached by the recent inflammation; bands could be distinctly seen when the membrane was lifted up. Some slight softening existed here, but none at other portions of the brain.

DR. STILLÉ commented on the interest of the case, which arose mainly from its being one of simple, non-tubercular meningitis, associated with tubercle of the lung. This is certainly rare, although by no means unprecedented. In what way the tubercular diathesis predisposes to meningitis it is difficult to say; yet that it does so appeared to him unquestionable. But in these cases we find, for the most part, small, granular, tubercular deposits along the base of the brain. No deposits of this nature, we are positively assured, could be found here by the most careful examination. We are, therefore, obliged to assume it to have been simple meningitis. Even some of the symptoms, such as the depression resembling that of typhoid fever, would seem to point to tubercular meningitis, yet the anatomical characters were not those of this affection.

DR. DA COSTA inquired if any effusion in the ventricles had been observed?

DR. MORTON. None.

DR. DA COSTA believed that this would tend to confirm the fact of its not being a case of tubercular meningitis. In this affection post-mortem examination almost invariably shows the presence of fluid in the ventricles, and, for the most part, in large quantities. The fluid is usually transparent and thin, but sometimes thick and creamy. It distends the ventricles and leads to softening of the surrounding tissue. Hence the softening in such cases is merely that from imbibition. The microscope bears this out; it reveals no evidences of inflammatory action.

*Case of Syphilitic Retinitis.*—DR. ADDINELL HEWSON read the history of a case of retinitis which he had considered syphilitic in its character, from its history and from the resemblance of the exudation on the retina to the condylomata of syphilitic iritis. He thought the case of some interest at the time he first saw it, and was not then aware of the fact of any similar ones having been observed. Recently, however, some cases of this very disease have been quoted in the various journals, as observed by Mr. Critchett, at the Royal Ophthalmic Hospital, Moorfield, London. As these have attracted a good deal of interest by showing the important *rôle* the ophthalmoscope is likely to assume in diagnosis, Dr. Hewson thought some details of the case which had come under his notice might be acceptable to the Society, and therefore read the following memoranda taken at the time, November 20th, 1858:—

Wm. M. Smith, aged thirty-one years, born in this country, a stout, hearty-looking man, with light hair and beard, and blue eyes, now weighs 190 lbs., but between two and three years ago his weight was as much as 234 lbs. At that time he contracted *primary syphilis*, for which he took some internal remedies, but does not know of what nature. Since then he has lost flesh, and his appetite has failed him. At one time his hair fell out in great quantities, and recently (within the last six months) he has had some sores on his skin, and has once in a while suffered with pains in his back and loins. He now complains of marked dimness of vision in the left eye, and says the right eye has also begun to fail him. He does not think there is any difference in his sight at any particular time of the day. He first discovered the defect in the vision of his left eye eighteen months ago. This discovery was made accidentally by his getting some dust in the right eye. He remembers, however, having had violent pain in his left temple some time previous to this accident. After this casualty he sought advice for his left eye, and was treated with blue mass, but this was not continued sufficiently long to affect his gums. The sight did not improve under this treatment. It now seems worse at times, and is perceptibly influenced by the condition of his digestive organs.

He first noticed his right eye being affected two weeks ago; since then he has had constant pain in the right temple. His appetite is now good, and he has recovered from his baldness. There is no apparent disease in either eye—no signs of his ever having had iritis, or any form of ophthalmia. With the ophthalmoscope, the lens and vitreous humor of the left eye are seen to be perfectly clear; the retina, however, appears to have a dirty tint, and to be defective in translucency; its surface is sprinkled all over with little points of a white, or a yellowish-white substance, and this effused substance in many places has quite a globular form, and resembles, in a very marked degree, the condylomata of syphilitic iritis; some of these masses have a reddish tint. The optic nerve in this eye has a dirty-red hue; the vessels of the retina are somewhat varicose. In the right eye the retina is seen to be more acutely inflamed; the optic nerve redder than natural, and the vessels of the retina, as they emerge from the nerve, abnormally large and tortuous; the tint of the retina is more dusky than is usually seen in acute retinitis.

I pronounced this case one of syphilitic retinitis, which had become chronic in the left eye; ordered Hyd. protiod, gr. $\frac{1}{4}$ t.d.; milk diet; rest; local depletion and counter-irritation. He improved for a time under this treatment, and was placed on the iodide of potassium. He was considerably benefited by this drug, which he took alternately with the protiodide of mercury. Whenever the gums and breath became affected by the mercury it was discontinued, and the potash taken in its stead.

Early in March his right eye appeared perfectly well, but no improvement was manifest in the left. About this time he exposed himself, when in the interior of the State, attending to some business, and became rapidly worse—almost blind in the better eye. The mercury was resumed, and, as soon as the acute symptoms had subsided, he again visited the city and consulted me. This was on the 19th of March, and I made the following note of this case at that time:—

March 19.—Wm. Smith visited me to-day; has improved much in health and appearance; says he weighed, two weeks ago, 200 lbs., but has lost since then; weighs now 195; has been suffering for two weeks with pain in both temples; sight is the same as when here on New Year's, but has been seeing, during the last two weeks, white stars,—these only occasionally; no external appearance of disease in the eyes; pupils natural; iris of right eye perhaps lighter in color; has been taking medicine up to the last two weeks; has had no flashes of light as he experienced after he was first attacked.

Dr. H. then ordered him the Zittmann's decoction; dysentery, however, supervened on the use of this, and his physician in the country was obliged to go back to the first plan of treatment. When Dr. H. last heard of this patient he was better, but not well.

*Report of Committee.*—The Committee appointed to examine the brain removed from a diabetic patient would respectfully report, that they carefully examined the organ, but found no portion which might fairly be considered as diseased. There appeared to be some softening in the immediate neighborhood of the fornix, yet this was a mere appearance, not borne out by a microscopical examination. The brain was very much engorged, and its tunics injected.

Signed DRS. PACKARD, HUMPHREYS, DA COSTA.

---

WEDNESDAY EVENING, April 28th, 1858.

The President, DR. GROSS, in the Chair.

*Cirrhosis of the Liver; Diabetes.*—DR. MORTON exhibited a specimen of cirrhosis of the liver, which was taken from a man aged thirty-eight, of short stature, thin, and dark-haired, who was admitted into the Pennsylvania Hospital on March 22d, 1858, for diabetes.

The account he gave of himself was, that with the exception of six or eight attacks of erysipelas of the right leg, he had been perfectly well until eight weeks before he entered the Hospital. At that time he was seized with another attack of erysipelas, and a severe cold followed; since which he had been confined to his bed. He stated that for a long time he had frequent calls to pass his water, and that he was obliged to rise five or six times during the night; his bowels were generally constipated; his tongue and skin, he had noticed, were always quite dry. For a long period he had been in the habit of carrying sugar in his pockets, and eating it while at work, so great was his desire for it; this habit had increased upon him of late. At the time of his admission he passed from seven to eight pints of urine daily, loaded with sugar, high-colored, and of a specific gravity averaging about 1038. His belly commenced to swell eight or ten days before he was admitted into the Hospital. The swelling had lately much increased, and he presented considerable œdema of the limbs and ascites, measuring thirty-eight inches around the belly. He became exceedingly debilitated and anæmic, and although he was placed upon a tonic plan of treatment, with rennet and nutritive diet, he improved but for a short period; he soon relapsed into his former weak condition, and his skin and tongue became again very dry. About the 12th of March he was seized with another attack of erysipelas in the leg, accompanied by diarrhœa; drowsiness supervened, followed by complete coma, and he died with symptoms of effusion in the brain, on April 16th, twenty-four days after admission, and eleven weeks after he first complained of being sick.

Post-mortem examination.—The lungs and heart were healthy; the liver cirrhosed, but of normal size; spleen, normal; kidneys, large, congested, and flabby; brain, pale; about two ounces of effusion were met with under the arachnoid. The abdominal cavity contained about $2\frac{1}{2}$ gallons of serum, which, when tested for sugar, gave evidence of the presence of a considerable quantity of this substance. The brain was examined for sugar, but not a trace of it could be detected. The blood from the ascend ing vena cava was also tested for sugar, but none was found.

DR. DA COSTA made some remarks with reference to the different organs which evince signs of disease in diabetes. The kidneys are sometims normal, but he had in several post-mortem examinations observed them to be larger than in health, and containing a much greater quantity of fat. The cells lining the tubules are filled with this substance, and the walls of the Malpighian corpuscles are full of oil-granules. Dr. Da Costa also directed attention to the circumstance that in most of the cases of diabetes presented to the Society, disease of the liver had existed.

DR. FORBES had met with disease of the liver in three post-mortem examinations made on diabetic patients. He also spoke of an interesting case of diabetes, which followed an injury. A patient, a negro, was brought into the Pennsylvania Hospital with a compound fracture of the leg. Diabetes made its appearance within a week, and he died of the disease in the thirteenth week after his admission.

*Tubercle in the Lungs and in the Mesenteric Glands.*—DR. MOREHOUSE exhibited two specimens of tubercle, one affecting lymphatic glands, the other the pulmonary tissue. In the first case disease had existed for more than three years. The glands of the neck had inflamed, softened, and opened, as is ordinarily seen in scrofula. This was followed by gradual emaciation, enlargement of the liver, and repeated discharges of blood from the bowels, which continued during the last ten months of the child's life. After death the mesenteric glands were found to be enlarged; so were the bronchial glands. The lungs were not the seat of deposit. The lower lobe of one of the lungs was in a state of inflammation. The heart was filled with firm heart clots, surrounded by a soft gelatinous mass soluble in water. The liver was greatly enlarged, and studding its surface were numerous subperitoneal deposits. Similar deposits were found on the spleen and pancreas, and on the inner surface of the abdominal walls. The microscopical appearances of the deposit in the different glands were those of yellow tubercle with a preponderance of granular matter.

In the second case the lungs were infiltrated with gray tubercle. A cavity had formed in one of them. The child, aged eleven months, died in convulsions. The ventricles were filled with fluid; their lining membrane was softened.

Dr. Morehouse believed that the first form, or yellow tubercle, affected more especially the lymphatic glands, and might be considered as a separate variety and as associated with the scrofulous diathesis.

DR. STILLÉ would not deny that tubercular matter was frequently deposited in lymphatic glands, but he did not think that this could be viewed as a separate form of tubercle. Nor could he regard it as identical with scrofula. For this disease, although chemically and in its minute structure not different from tubercle, was yet very dissimilar in its exact clinical and pathological bearings, and a distinction, therefore, between the two is being now more generally admitted.

Several other members maintained similar views and spoke against Dr. Morehouse's proposition.

*Cyst over the Nasal Bones containing Gelatinous Matter.*—DR. GROSS exhibited a cyst removed from Albert B. M., aged eighteen years, who presented himself at the Jefferson College Clinic, on account of a tumor which had existed for sixteen years over the nasal bones between the inner canthus of the left eye and the glabella. Upon removal, the tumor, which was about the size of a marble, was found to contain a small quantity of softened sebaceous matter, and about one drachm of a greasy, light, amber-colored fluid, resembling in every respect fine olive-oil. This, upon standing a few moments, became of the consistence of lard. The cyst was opaque and thickened, and granular on its inner surface. Microscopically, the fluid was found to consist of fat globules and numerous non-nucleated corpuscles, such as are seen in the gelatinous matter taken from the thyroid gland.

*Astragalus discharged through an opening in the Foot.*—DR. HUTCHINSON presented, through the Secretary, an astragalus removed from the left foot of Josiah Morgan, aged eleven years, of Butler, Luzerne County, in this State, by Dr. Peace, at the Pennsylvania Hospital, on the 17th of March.

The patient states that, about the middle of last August, he was thrown from a hay-mow by another boy, but not feeling much hurt he continued to use his foot for some days afterwards. During this time he caught his foot in a rut and twisted it slightly; he is unable to say positively to which accident he owes his present condition. A few days after the latter occurrence his foot became too painful to be used. There was no external wound at this period; an opening formed, however, a short time subsequently. Several small pieces of bone were discharged before his admission into the Pennsylvania Hospital, and the remainder, constituting the entire astragalus, was removed without difficulty.

When the case was first seen, a short time after admission, there existed in front of the external malleolus a large opening into which the little finger could readily be passed. This has now healed, leaving only a small orifice, through which carious bone may be felt. Another and similar orifice existed lower down, over the os calcis.

The principal shortening took place in the length of the foot, it being now three-quarters of an inch shorter than the other; not more than one-quarter of an inch difference can be detected in the length of the legs.

The foot had a tendency to turn inward, which has been corrected by a pasteboard splint moulded to the part. The foot is very nearly at the proper angle with the leg, and some motion exists at the joint.

---

WEDNESDAY EVENING, May 12th, 1858.

The President, DR. GROSS, in the chair.

*Cases of Chronic Rheumatic Arthritis.*—DR. BRINTON exhibited two shoulder joints which had been removed from a subject in his dissecting room. These specimens presented appearances such as would lead to the belief that, during life, the individual had suffered from chronic rheumatic arthritis.

*Examination of left shoulder.*—The upper and outer portion of the capsular ligament had been entirely destroyed. The tendon of the biceps muscle had been absorbed; the tendons of the supra and infra-spinatus, and also that of the teres minor, had become separated from their attachments to the tuberosities of the humerus; and that of the last-named muscle was completely torn into shreds. The synovial membrane of the interior of the joint was thickened and presented a villous fimbriated appearance. In consequence of the destruction of that portion of the capsular ligament which arises from the upper part of the glenoid cavity of the scapula, a spontaneous luxation of the humerus directly upwards had occurred; the head of the bone resting against the under surface of the coraco-acromial arch. The articular cartilage of the humerus in some points appeared thin and soft; at others, and especially upon the outer and upper portion, eburnation had commenced, as also upon the under surface of the acromion process.

*Right shoulder.*—In this preparation, which had been cleaned, the same appearances of eburnation were presented, and destruction of the capsule and upward dislocation had also occurred. Dr. Brinton stated that some time since an opportunity had been afforded him of examining upon the living patient the interior of a shoulder-joint similarly affected. The patient, aged twenty-three, had suffered from repeated attacks of chronic rheumatism. Some months subsequent to his last attack a tumor developed itself slowly in the region of the supra-spinatus fossa of the left shoulder, producing pain, and interfering with the utility of the limb.

Under the impression that the tumor was of a fatty nature, an incision was made over it; when about two ounces of dark, thick, oleaginous fluid escaped. The supra-spinatus muscle was found completely destroyed, having undergone fatty degeneration; its tendon was separated from its belly, and had formed attachments to the bone. The finger could be carried under the acromial arch into the cavity of the articulation, where the capsule appeared to be destroyed externally and superiorly, and the head of the humerus seemed rugged and uneven. The degenerated supra-spinatus muscle was scraped away with the handle of a knife, and the wound closed by sutures. Union by first intention ensued except at one point, where a synovial fistula existed for some weeks, but eventually healed up.

The patient recovered the use of his arm and was able to resume his work, but died of a cardiac affection some months afterwards. Dr. Brinton stated that he regarded this case also as a well-marked instance of the rheumatic arthritis so ably described by Mr. Smith and Dr. Adams, of Dublin.

Dr. Brinton also directed the attention of the Society to a specimen of remarkable *disease of the knee-joint.* The patient was a negro wood-sawyer of advanced age, but the exact history of the case could not be obtained. The joint exhibited presented the following appearances:—The crucial ligaments had been destroyed, as also the semilunar cartilages and coronary ligaments. The external condyle of the tibia had been absorbed and a new articulating surface formed at the expense of the side of the tibia and the head of the fibula; this latter was flattened out and incrusted with bony deposits. The adventitious cavity thus formed, as well as the condyle of the femur, had become completely eburnated, and presented surfaces of extreme polish and hardness. In the new condyloid cavity was found a large mass of bone attached by a ligamentous pedicle to the external ligaments. This mass was about an inch and a quarter in diameter, of rough and irregular shape, although at certain points polished by attrition. As long as this body rested beneath the external condyle of the femur, and between it and the bottom of the new articulating cavity, it was evident that the patient could walk with the leg nearly in its normal relative position to the thigh. When, however, the mass of bone slipped away from beneath the femur, as frequently had happened during life, a spontaneous dislocation of both bones of the leg occurred, the leg then forming with the thigh an angle of about 135°. Two or three free cartilages, or rather osseous masses, were found loose in the joint. The synovial membrane had been entirely destroyed. The external ligaments had lost their distinctive character; the whole articulation being surrounded by a species of dense capsular ligament. Fracture of the patella had also occurred; the upper fragment had been displaced laterally, and the union was imperfect. As to the origin of this strange condition of the joint, Dr. Brinton could not speak with certainty; but he was inclined to look upon it as the result of chronic inflammation, perhaps following injury.

DR. HEWSON was able to add some facts concerning the history of this patient. The man had earned at times his living by cutting grass, and had been thus occasionally employed by Dr. Hewson's father. Dr. Hewson could state positively that he had suffered much from chronic articular rheumatism. He also remembers distinctly that fourteen or fifteen years ago he met with an accident to his leg, probably a fracture, after which his lameness was much increased, and that at times this man walked more erect than at others.

*Constriction of the Mitral Valve; Single Kidney.*—DR. MORTON exhibited a diseased heart and a fused kidney removed from a patient who was admitted into the Pennsylvania Hospital on May 3d, 1858, about 6½ P.M. The only account which could be gained of the case was that the man was found in an insensible condition in one of our small streets. On examining him, it was soon perceived that he was in a violent convulsion,—skin cold, eyes staring, great muscular rigidity, and the pulse scarcely perceptible. He was immediately placed in a hot bath, 110° F., and carbonate of ammonia and brandy were given to him and were readily swallowed. Thinking that possibly some poison had been taken, the stomach-pump was introduced and the viscus washed out; but there was no evidence of any foreign substance. He continued to sink, his pulse sometimes rising and then falling, until 1½ A.M., May 4th, when he died, exactly seven hours after admission.

Post-mortem examination twelve hours after death.

Lungs, healthy. Heart, valves healthy, with the exception of the mitral, which was much thickened, and so contracted that it would not admit the little finger; large fibrinous clots occupied all the cavities. Liver, large and fatty. Kidneys, presented the following anomaly: the two kidneys were merged into one, which was placed near the third lumbar vertebra, and so situated, in relation to the spine, that about four-fifths of the organ were on the left side, and the remaining portion on the right. That part of the kidney which lapped over the bodies of the vertebræ was much thinner than the main body. The organ has an irregular kidney shape; on the back surface of the gland the parenchyma is deficient in two places, one near the right, the other near its left extremity.

The cavity at the left, which is connected with the main body of the kidney, measures about an inch and a quarter in length, and three-fourths of an inch in breadth; the right cavity is about three times the size of the left, and is of an irregular, quadrilateral shape, and much more superficial than the other. These two cavities contain the pelves of the kidney. The right portion of the kidney sends six calices to the pelves; the left pelvis is pyriform in shape; there is no communication between the two pelves. One artery supplied the organ, sending two branches to the left and one to the right side. The kidney measures six and three quarters of an inch in length, four and a quarter in breadth, two and three-quarters above the contracted portion. The left ureter is twelve and a half inches, and the right ten inches long. On leaving the kidney, they pass down on the right side of the spine, and enter the bladder at the usual places. The organ weighs nine and a half ounces; in consistence it is healthy.

The Brain weighed sixty-three ounces. It was quite soft, and easily broken down when pressed with the finger. In many places it presented the appearance of chronic congestion, particularly on the right side, and nearest the cerebellum. The membranes (arachnoid and pia mater) were much thickened, opaque, and filled with serum; about eight ounces of which escaped when the brain was removed.

In the falx cerebri, about a quarter of an inch from the lower boundary, and two inches from the anterior edge, was found an irregularly spiculated piece of bone, sharp and pointed; several other much smaller pieces were seen at some distance from it. The arteries of the brain contained large, firm, fibrinous clots, which could be drawn out continuously for some inches.

When the calvarium was removed, a depression in the brain was observed, and, upon searching for the cause, a bony outgrowth from the frontal bone was found, which projected inwards. It was situated above the right frontal sinus; other small bony projections were to be seen near it.

It is most probable that the brain suffered so much from the pressure of the tumor, that epilepsy was the result; the condition of the membranes would also favor this supposition.

DR. GROSS inquired into the state of the supra-renal capsules.

DR. MORTON.—One existed.

DR. LEVICK commented upon the mode in which death had occurred in this case, and remarked, in this connection, that the fibrinous clots extending from the heart into the smaller vessels of the brain, might have a direct bearing in explaining the symptoms preceding death.

DR. RICHARDSON alluded to the rarity of a single kidney.

DR. GROSS stated that he had a specimen of this kind in his collection.

DR. HARRIS presented the following remarks in relation to the specimen before the Society:—

The human kidney is subject to numerous variations in regard to its position, form, mobility, vascular connections, and the arrangement of its excretory ducts. In some instances one, and in others, both glands have been found wanting; but those in which the latter condition is said to have existed (except in the cases of newly-born infants) are not sufficiently well authenticated to be received as positive proofs. Two, and sometimes more, kidneys are occasionally united to form one; and numerous examples of this error of conformation may be found by referring to the medical periodicals and standard works which have been issued during the last two hundred years. In the journals of our own country, very few cases have been reported. "The American Journal," with its predecessors of the same series, running through a period of fifty-four years, does not report a single case; the same may also be said of "Bell's Medical Journal;" "The Boston Medical and Surgical Journal;" "Philadelphia Medical Examiner;" "New England Journal of Medicine and Surgery;" "North American Archives of Medical and Surgical Science;" "Virginia Medical and Surgical Journal;" "New York Journal of Medicine;" "Boston Medical Magazine;" and "The Transylvania Journal of Medicine and the Associate Sciences."

Before entering more particularly upon the subject of *fusion* of the kidneys, it may be well to examine into the nature of the other errors of conformation and position, above referred to.

1. *Position.*—This is quite variable, one or both glands being not unfrequently found higher or lower than the situations in which they are usually placed. Many cases are upon record, where one or both of the kidneys (though seldom the latter) have been found in the superior or inferior pelvis; and instances of the first variety have no doubt occasionally been the means of leading an examining physician to conclude that his patient had had but one kidney. In an example recorded by Rayer,* of which a plate may be found in the Atlas accompanying his work, the misplaced kidney was discovered low down in the pelvis, in front of the sacrum, and in a vertical position; it was smaller than the one normally situated, and received its arterial supply from a vessel which was given off by the aorta, between the origin of the primitive iliacs. As the other kidney was larger than natural, and the capsula renalis of the misplaced one in its usual situation, the latter might very readily have escaped the eye of a physician in making a post-mortem examination, if conducted, as we are frequently obliged to conduct them, in private practice. In most cases of

* Maladies des Reins, tome iii. page 784.

misplaced kidney, the supra-renal capsule of the same side occupies its accustomed seat, thus giving, by its independence of position, one proof of the absence of any material connection in the functions of these two bodies. Besides the forms of misplacement mentioned, cases have been met with where the two kidneys were discovered lying in front of the sacro-iliac symphysis, or placed close to each other, and in front of the vertebral column. The *floating kidney*, hereafter to be referred to, is another example of malposition.

2. *Size.*—This is subject to more variation than the position, though often influenced by the latter. Disease is one great cause of augmentation or diminution of the gland; but with it we have nothing to do at present, having only reference, in this connection, to variations in volume, which result from other causes. When one kidney is smaller than natural, the other is usually found correspondingly enlarged; and where there has been an early arrest in the development of one, the other is nearly or quite double the natural dimensions. The same may also be said of the effect of the absence of one kidney. Single kidneys seated in the lower pelvis are generally smaller than natural, and have a very short ureter.

3. *Form.*—Single kidneys vary but little in shape when in a healthy state, except when their growth has been affected by the peculiar position in which they have been placed. A few instances are upon record where a single kidney was found longer and much more curved than natural, constituting one of the varieties of *horse-shoe kidney*, though this title is generally applied to a peculiar variety of fusion. Kidneys developed in the pelvis are generally of a different form from those found in the lumbar region, being usually more cylindrical and narrow; though in this there is no uniformity, as the shape depends very much upon what part of the pelvis they occupy. We shall have more to say upon the varieties of form, when we come to consider more minutely the subject of fusion of the kidneys.

4. *Mobility.*—In their natural position the kidneys are very firmly fixed, but we sometimes meet with cases in which the arteries and veins connected with a single kidney, or with a fused one composed of two, are exceedingly long, and act, to a certain extent, as a means of suspension for the gland, allowing it to float, as it were, among the intestines, in the abdominal cavity. Such cases are sometimes mistaken for abdominal tumors, and are very difficult to recognize in the living subject. Though not properly a disease, this peculiar condition may give rise to much disturbance of the digestive organs. An example of this malformation may be found in the work of Rayer, before quoted; another in the "Boston Medical and Surgical Journal," vol. xxv. page 275; and four in the "London Lancet" for 1842–3, vol. ii. page 558. The last were observed by M. Aberle, and reported by him; one of them is taken from the "Salzburg Gazette," of 1826, and the other three from "Schmidt's Jahrbuch," vol. xxxii. p. 312.

5. *Vascular connection.*—The kidneys, in their natural form and position, are generally each supplied with blood through one arterial trunk, and the circulation returns through one vein; but various departures from this rule are to be met with; sometimes there are several emulgent arteries to a single kidney, and when a fusion of two or more glands has taken place, it is common to find for each section a separate artery and vein. In misplaced kidneys the renal artery may come from some point considerably below the natural level of the kidneys. In floating kidneys, however, the veins and arteries are usually given off from the points whence they proceed, in cases where the glands are normally situated.

6. *Arrangement of the excretory ducts.*—Variations from the usual condition are not at all rare. A single kidney may have a double pelvis, or two pelves and two ureters, which last may either unite to form a common trunk, or continue separately to the bladder. Rayer gives a plate of one case where each kidney had two pelves and two ureters, and these conduits all entered the bladder distinct from each other. The ureters from two kidneys may unite to form one common canal. In fused kidneys there may be one, two, three, or four ureters, which may continue distinct from each other until they enter the bladder, or two or more may unite to form a common conduit. The ureter may not enter the bladder at all, but empty into the urethra; a case of this character, where the ureter was double, and entered the urethra just in front of the neck of the bladder, may be found recorded in the "Boston Medical Magazine," vol. iii. page 332. In fused kidneys the calyces are generally much longer than natural, and are exposed to view by simply removing the fat which covers the depression in which they and the pelvis are situated. We now come more particularly to the subject of union of the kidneys, and with it may properly be included that of redundancy of kidneys.

Though comparatively of rare occurrence, fusion of the kidneys has been met with sufficiently often, during the last two centuries, to have given, in the medical books and periodicals where they have been reported, a considerable number and variety of examples. The most common form of union is that known as the *horse-shoe kidney*, (this title, as I have before mentioned, is sometimes also given to a single kidney of an unusually curved form,) in which the two kidneys are united across the spine at one or other of their extremities, (in most of the instances I have found recorded it was the lower,) either by a direct union, or through the medium of a connecting band or *isthmus*. Another variety is that in which there are more than two kidneys united together in a somewhat crescentic form, and showing, by constrictions at certain points, the number of glands composing the whole mass. Each section is supplied with its own vessels, and sends off a separate ureter. I will refer to a few of the cases which have been reported, as examples of these peculiar forms of fusion.

Rayer* reports, with an accompanying drawing, a case of horse-shoe kidney, with the concavity upwards, having a double pelvis and a single ureter. One instance of the more common horse-shoe kidney, with two ureters, such as they are usually found, is reported in the "Dublin Quarterly Journal of Medical Sciences," for 1846, vol. ii. page 239; another in the "Edinburgh Medical and Surgical Journal," for 1819, vol. xiii. page 90; and three cases in the "London Lancet." Similar cases have been met with by Panthot, Kaltschmidt, Bartholin, Wrisberg, Gebbard, Sandifort, and many other European writers. Rayer mentions having seen a crescentic kidney in which there were three distinct glands, united by their extremities. Botal, Gemma, Dalestang, and Monginot report cases of quadruple kidneys, each section of which possessed its distinct pelvis, ureter, and blood-vessels. In all these cases above referred to, the fused kidney was situated either transversely or obliquely across the spine.

From all the information that I have been able to collect from the monographs and journals consulted, I am led to the conclusion that the variety of double kidney exhibited by Dr. Morton is, with one exception, the most rare of all the examples of fusion of the two glands. The exception referred to, is that form of double kidney described by Sandifort,† where one gland was found in its natural position, and the other placed immediately in front of, and united with it; two pelves and two ureters proceeded from this welded gland.

I have been able to find but two cases recorded in all the volumes of journals, (amounting to several hundreds,) and in the books that I have examined, which very nearly resembled the specimen now before us. One is delineated in Rayer's plates, and the other is reported in the "North American Medical and Surgical Journal," of 1829, vol. vii. page 176; a reprint from a foreign periodical. Both these kidneys were seated transversely across the lumbar vertebræ, and had two pelves and two ureters, which, in each specimen, came from the anterior surface of the mass.

Three peculiarities distinguish the fused kidney upon the table from all those of which I have found full descriptions. 1st. Almost the entire mass was situated upon one side of the spinal column, a small, thin portion only, overlapping the vertebræ. 2d. The concave edge, instead of the convex, presented downward, and the pelves toward the psoas magnus

---

* Op. Cit.

† Obs. Anatom. Pathol., Lib. iii. Cap. vii. page 96.

muscle. In most instances the pelves are either upon the abdominal surface or the concave edge of the gland. 3d. Although the gland, from its size and the number of its ureters, is clearly a double one, in form, position, and vascular connection, it bears a close resemblance to a single hypertrophied kidney.

*Cancer of the Lung and of the Mesenteric Glands.*—DR. DARRACH presented a cancer of the lung, and remarked as follows:—

The subject of the above-mentioned disease was a married man, aged sixty-five years. He was of a spare figure, had a well-developed chest, and could count with ease 100 in a breath. At the time he was first examined (Jan. 6th, 1858,) no affection of the lung could be discovered, by a careful physical examination of the chest: the symptoms of which he complained were vomiting of food, acidity of the stomach, eructations, and, on pressing over the region of duodenum, a "deep soreness." His indisposition first appeared about two years ago, and had increased most rapidly within the last three months; but more especially for the last two or three weeks. Within the latter space of time he retained scarcely anything on his stomach. He had also become lately very irritable in temper, and indisposed to use either body or mind. Thinking that the patient might be suffering from a severe attack of mal-assimilation, commonly called oxaluria, a specimen of his urine was examined, but no oxalates were discovered. The quantity of urine passed in the twenty-four hours was about forty fluidounces, (sp. gr. 1020.) In less than forty-eight hours, in a cool room, the fluid still remaining slightly acid, there appeared a most abundant formation of vibriones, which constituted a dense cloud throughout the mass of urine. From this formation it was inferred that he was laboring under some very depressing influence, the nature of which was not as yet perceived. The patient had also been suffering for some weeks from hemorrhoids.

The treatment followed out at this time was as follows:—Nux vomica, gr. $\frac{1}{8}$ four times daily, with blue pill at night, and carefully regulated diet, &c. But the patient continued to grow worse and worse. Emaciation became extreme; the hemorrhoids, for some weeks before his death, bled at every discharge from the bowels, frequently as much as a pint. His mind became at times wandering, and he seemed to be succumbing, as his family physician remarked, to some serious blood-disease. A short time previous to his death, cancer was surmised, probably of the liver, yet the pulmonary symptoms remained extremely obscure. There was no hemorrhage, and but a trifling cough.

Death occurred on the 26th of March. An examination of the body was made on the afternoon of the 27th. The cranial cavity was not examined. The upper lobes of both lungs were solidified with what appeared to the naked eye to be undoubted tubercle, but which, on careful examination with the microscope, by Dr. Da Costa and myself, was satisfactorily proved to be cancer. The elements found consisted of cells with large nuclei, many of the cells being peculiarly transparent, and exactly resembling those described by Dr. John Hughes Bennett, in his work on "Cancerous and Cancroid Growths," page 45, as occurring in cancer of the lung. There were also cancer nuclei and granular matter.

The liver was dotted over its surface with very small, perfectly circular patches, of a white matter, dipping slightly into the liver substance, their centre having a depressed appearance, as is not unfrequent in cancer of that organ. I could not satisfy myself as to the cancerous nature of the elements composing them.

The stomach, bowels, kidneys, and spleen were normal; the lumbar glands were very much enlarged by a deposit, which gave to them the appearance of scirrhous cancer, yet on cutting into them, they were neither very hard nor resistant. The cut surface became convex, and was of a slightly yellowish hue; no creamy fluid exuded. Microscopically examined, these glands showed elongated, nucleated, fibroid cells, such as Paget has described as occurring in some structures affected with medullary cancer.

DR. STILLÉ inquired of Dr. Darrach whether diarrhœa had existed, and also what was the state of the glands in the small intestine?

DR. DARRACH.—The glands were not examined; diarrhœa had not been a symptom during life.

DR. STILLÉ spoke of the absence of diarrhœa as being a symptom of importance as regards the diagnosis between cancer and tubercle of the mesenteric glands. He believed that in an adult, tubercle of the mesenteric glands is very rarely, if ever, met with, without the glands of the intestine being affected. As here no diarrhœa existed, and as the signs of disease of the lung were evidently consecutive to those of the abdominal cavity, he thought the presumption, even during life, would have been strongly in favor of cancer.

*Cancer of the Pancreas and Stomach.*—DR. AGNEW presented a specimen of medullary cancer of the pancreas, with a similar deposit at the cardiac orifice of the stomach. The patient from whom they were removed (aged about fifty-six years) came under the care of Dr. Ludlow nine months ago, and from the character of the symptoms, the case had been diagnosticated as one of cirrhosis of the liver. The patient's first indisposition was three months previous; up to this period he had enjoyed good health. He was a good liver; fond of hot suppers and richly cooked food; his hours of eating were irregular, sometimes very late at night; he used malt drinks habitually, and occasionally spirituous liquors, but not to any great extent. His weight at the time of his attack was 250 lbs. The first symptoms of indisposition were those of ordinary indigestion, with occasional nausea and oppression at the stomach, a feeling of fulness over the abdomen, and constipation of the bowels. The loss of a situation, which he had held for several years, depressed his spirits very greatly; and with this his physical distress increased rapidly. Vomiting occurred; pains of a flying character passed through his abdomen, chest, and right shoulder; the stomach rejected for a time all kinds of food; the matter vomited was of a glairy, viscid character, and exceedingly offensive; hiccough set in, and became very troublesome; also great accumulation of gas occurred, which could only be expelled by thrusting the fingers down the pharynx, in order to produce forcible gagging. His strength failed, and emaciation progressed rapidly. At one time nothing seemed to arrest the hiccough so well as small pieces of hoarhound candy; this, however, soon lost its effect, and the continued hiccough became very distressing. During the intervals of repose he would allow no one to move, shut a door, or even speak, as the least excitement would produce a violent return. The hiccough was finally controlled by small doses of strychnia and morphia. The vomiting and the unpleasant gastric symptoms were much benefited by a combination of ext. taraxici with an alkali and creasote, and by occasional small doses of calomel and bismuth. His complexion became pale and bloodless, and his countenance distressed; the eyes anxious; the pulse small and feeble. The bowels were very torpid; when moved, the evacuations were clay-colored, but sometimes of a greenish hue; the urine was high-colored, and deposited a heavy sediment; it contained bile and much mucus. Though latterly he was very much relieved of nausea and hiccough, yet emaciation went on rapidly; the fat disappeared almost entirely, and he appeared to die from entire want of assimilation.

Autopsy confined to the abdomen.—The body was reduced to about 120 lbs., its color indicating a cancerous cachexia. On turning off the abdominal walls, the tissues and organs were found to be perfectly blanched from want of blood; no peritonitis existed; the liver was in a complete state of cirrhosis, also enlarged; the gall-bladder empty; the kidneys were in a state resembling cirrhosis; the pancreas increased to more than four times its size, its glandular structure having apparently disappeared, and being replaced by a mass of brain-looking substance; the stomach, when opened, seemed to be healthy, except at the cardiac orifice, where a deposit similar to that in the pancreas existed. On examination by the microscope, both in the deposit in the stomach and pancreas, cancer-cells were found, remarkable for their great size and diversity of form. The rapid emaciation Dr. Agnew attributed to a mechanical as well as a constitutional cause, namely, pressure of the pancreas on the thoracic duct.

*Aneurism of the Aorta, bursting externally.*—DR. PACKARD exhibited a specimen of aneurism of the aorta, and remarked as follows:—The patient, a colored man, aged forty-eight, was a porter in a carpet store, and kept at work until the latter part of February last.

In July, 1857, he had a cough, which, however, was not very severe, and soon afterward he perceived a swelling at the upper and front part of his chest. He applied to me in February, 1858, (seven months later,) with a swelling of the size of a large fœtal head, occupying the site of the upper part of the sternum, and extending more to the right than to the left of the median line. The pulse in the two carotids was almost alike,—that in the right radial was slightly less than that in the left. There was no numbness, but some pain, in the right arm, and a very marked pulsation in the tumor, but *no thrill whatever;* the clavicle on each side seemed bent and pushed forward, carrying the sterno-cleido-mastoid muscle along with it.

The treatment adopted was palliative; but the tumor enlarged so rapidly as to measure, on the 2d of May, 10¼ inches in semi-circumference from one side to the other, and 11 from above downward. Discoloration appeared at the most prominent part, and on the 6th of May, at 10 P.M., a small slough separated; some bleeding ensued, which was checked by means of a hemlock plaster till 6 A.M., on May 7th, when it recurred so violently that his death was instantaneous.

Autopsy 10½ hours after death.—The tumor only was examined; its anterior wall and the superjacent skin were extremely thin, and closely adherent to one another; they could, however, be separated with the fingers, except where traces existed of the sterno-cleido-mastoid muscle, and here the union was very close and firm. The sac being cut into, an immense clot was found in it, very firm below and in front, as well as at the posterior part, but becoming grumous above, toward the point where the fatal opening had taken place. Several ribs were felt projecting into the cavity of the sac on each side, in a roughened state, and a portion of the sternum lay imbedded in the clot, in a similar condition. The vessel involved was the ascending aorta and the arch; the innominata was increased in diameter to about an inch; the left carotid and subclavian were normal. Atheromatous deposits existed, to a great extent, in the descending aorta. The trachea was flattened, and pushed over to the left side of the vertebral column, which was sound; the lungs were healthy, but adherent to the tumor by their apices.

DR. DA COSTA stated that, through the kindness of Dr. Packard, he had had an opportunity of examining the patient during life, and it had struck him, as a point of great interest, that not only was no thrill present, but also no blowing sound could be perceived. The sounds over the tumor were two sounds like those of the heart. The second sound was short and indistinct; the first was long and prolonged, and, if such an expression be permissible, weighty. They differed not only in strength from those of the heart, but also, it had seemed to him, in pitch; this was especially the case with the first sound. Both sounds of the heart, listened to at the apex, and ensiform cartilage, and at midsternum, near the aortic cartilage, were distinct, but not otherwise altered. The impulse of the heart was forcible, and somewhat extended.

DR. STILLÉ inquired into the probable production of these sounds in the tumor. Were they not produced in the sac?

DR. DA COSTA could not answer this positively. As the sounds were stronger than those of the heart, they could not have been transmitted sounds, unless we assume that they gained in strength by the peculiar physical conditions of the sac. If not thus produced, they must certainly have been generated in the sac.

DR. PACKARD did not believe that they were thus caused. He regarded the sounds heard over the tumor as the transmitted heart-sounds, since their similarity was very great.

*Urinary Calculus.*—DR. ALBERT H. SMITH presented, through the Secretary, a stone weighing 164 grains, which was extracted from the bladder of a child two years old, by Dr. Norris, in the Pennsylvania Hospital. The following history accompanied the specimen:—George Grater, aged two years, of Bucks County, Pa., was admitted into the hospital on the 3d of May, 1858. He has a light complexion, blue eyes, light hair, and looked quite anæmic, and very delicate and sickly; his countenance exhibited marked distress, presenting, for a child, a most care-worn expression. From the day of his birth he has suffered with incontinence of urine, the discharges being interrupted, and attended with great pain, and with excessive tenderness in the whole perineal region. He had great nervous irritability, deficiency of appetite, restlessness, morbid fear of strangers, fretfulness, and every symptom of a constitution worn and harassed by constant internal irritation. His parents stated that he has not passed a day since his birth free from acute suffering.

When admitted into the hospital he exhibited the same symptoms as above mentioned, besides having prolapsus ani and diarrhœa. By sounding, a calculus was detected in the bladder. On the 5th inst., Dr. Norris removed from this viscus, by the lateral operation with the gorget, a phosphatic stone weighing 164 grains. Notwithstanding the unfavorable condition of the child previously to the operation, he has not had an unpleasant symptom since. His restlessness and general irritability have greatly diminished; he sleeps well, has a good appetite, and, as his mother says, he seems more comfortable than he has ever been since his birth.

The most interesting feature of this case is the evidence of the affection being congenital. Cases of stone in new-born children are rare, so far as statistics show, though enough are recorded to prove their possibility. Civiale expresses himself entirely free from doubt on this point; and, although he has never seen a case himself, he quotes from Brendel a case of a child who passed a red sand, and dying on the second day after birth, its bladder was found to contain a stone of considerable size; also two cases of children in whom calculi were found in the fossa navicularis within twenty-four hours after birth. Numerous instances are also cited in children, of age ranging from two days to six months, in which there was probability of the stone having been formed in utero. That this affection is rare, however, in infants so young, may be inferred from the fact, that out of 5376 cases collated by Civiale there were only three under one year of age. One case mentioned as having been operated upon at that age was followed by death. The mortality in thirty-seven cases operated upon at two years of age was seven.

The size of the stone extracted from this boy is much greater than any that I have been able to find recorded as met with in a child of two years of age. The largest one mentioned at that age weighed one drachm, and is quoted by Crosse. It occurred at the Norwich Hospital.

---

WEDNESDAY EVENING, May 26th, 1858.

The President, DR. GROSS, in the chair.

*Dilatation of the Meningeal Artery, producing a Bony Protuberance on the Head.*—DR. AGNEW exhibited a skull-cap which had, on its internal surface, a very large groove for the meningeal artery. This groove terminated in a dilatation near the vertex, probably of an aneurismal nature. At the seat of the groove the internal table was absorbed, and the external table formed a considerable protuberance. The artery on the other side was large, but in its dimensions far inferior to that of the left side. The specimen was obtained in the dissecting room, and no history could be procured.

DR. STILLÉ would ask of Dr. Agnew whether he thought that this protuberance could, during life, have been distinguished from an exostosis, and its cause determined?

DR. AGNEW thought that a careful examination might have detected a thrill.

DR. STILLÉ had reference to this in asking the question. He also believed that a thrill might have been present, and thus served as a diagnostic sign. This aneurismal dilatation is extremely interesting. The occurrence of a tumor of the kind might lead to the erroneous supposition of a syphilitic exostosis.

DR. AGNEW believed that exostosis, syphilitic or otherwise, most frequently springs from the inner table, which, on the contrary, in the specimen before the Society, was absorbed.

DR. HARRIS did not agree with Dr. Agnew in this respect. He had a head in his possession, where, from a fall, an exostosis of the outer table had occurred.

DR. HEWSON also differed from Dr. Agnew with reference to this point. He had now a young lady under his charge in whom an exostosis had been developed after accidently striking her head violently against the door of a carriage. The protuberance was evidently produced by deposit on the external table. There had been no fracture, and there was no pulsation nor thrill to be felt, although the patient herself experiences a pulsation at times in the tumor. Dr. Hewson alluded also to the fact that pulsating tumors on the scalp, supposed to be aneurismal, might be of a malignant nature, as an interesting case reported by Dr. Neill proves.

DR. GROSS had seen several cases of pulsating tumors on the head, dependent upon dilated arteries. Two in especial were interesting. They were both of several years' standing, and led to absorption of the bony tissue, bringing thus the scalp and the dura mater in contact.

*Bright's Disease—Fibroid Degeneration of the Kidney.*—DR. MORTON presented a kidney filled with fibrous deposits. It was taken from a man who died in the Pennsylvania Hospital from effusion into the brain. Prior to his admission he had received a compound fracture of the leg, for which his limb was amputated at Norristown. The man, after being conveyed to the hospital, became comatose, and sank rapidly.

Urine taken from his bladder after death was examined, and found to contain granular casts. The kidneys, when cut into, presented a glistening whitish appearance, and were very firm in consistence. Microscopically examined, they abounded in fibrous tissue and fibroid cells.

DR. HEWSON spoke of the prognosis of amputation always being more unfavorable in cases in which the urine contained albumen, or showed, as pointed out by the observations of Bence Jones, signs of waste of tissue in the economy.

*Imperforate Rectum.*—DR. STILLÉ, on behalf of DR. J. FRANK BELL, presented the following notes of a case of *imperforate rectum:*—

*Past History.*—The mother of the child, healthy and multipara, was delivered at full time on May 11th, 1858. The child was of good size, and appeared healthy; but on the third day, no meconium having passed, I saw it for the first time, and made an examination, when the following appearances were presented:—

A well-formed male child; seems healthy, and is tranquil; urine clear and passed freely; anus perfect; admits a probe three-fourths of an inch, when it meets with firm resistance; on very slight pressure against the probe the child cries.

*Diagnosis.*—The most simple form of imperforate rectum. An operation was deferred on account of there being no convexity of the membrane downwards.

May 15th, 2 P.M. Saw the child and made a second examination. The parts present the same appearance as yesterday. An old-fashioned ear speculum showed a well-formed *cul de sac*, with folds of mucous membrane at the bottom. There is still no protrusion, or convexity, of the membrane; abdomen tense in hypogastric region. Assisted by my friend, Dr. E. R. Duval, I punctured the membrane across the rectum with a medium sized trocar; after its introduction the stylet was withdrawn, when meconium passed through the canula. The trocar met with considerable resistance. The removal of the canula was followed by the discharge of one or two fluidrachms of blood of a bright-scarlet hue. The bleeding, however, stopped after a few moments, when the child was put to rest, and a dose of sweet oil given.

May 16th, 12 M. Some meconium passed last evening; also a small quantity this morning. The child rested badly last night; appears hungry, but refuses the breast; abdomen slightly enlarged; tympanitic. A medium sized catheter is introduced into the rectum with ease, but seems to be grasped at the point where the perforation was made, so that it turns on its axis with difficulty. An injection of warm water was thrown into the bowel, and was expelled with considerable force, accompanied by meconium and flatus; repeated injections procured a tolerably free discharge of meconium. Sweet oil was again administered.

May 17th, 2 P.M. No meconium passed since the last visit. The child rested well last night; nursed once in the fore part of the night; vomited twice in the latter part; vomiting not stercoraceous. The nurse thought it dying this morning at 10 o'clock. It, however, soon rallied; but has been growing gradually weaker since. Abdomen quite tympanitic; the child is very weak. The tube passed readily into the bowel, but is firmly grasped at the seat of the artificial opening; warm water injection used, which was expelled, accompanied by meconium and flatus; some toddy administered, after which the child rallied, but soon again began to sink, and died at 5 P.M.

May 18th, 7 A.M. Post-mortem examination twelve hours after death.—

Abdominal viscera normally situated; extensive peritoneal inflammation; mucous membrane of stomach and intestines healthy down to the rectum; the lining membrane of the latter highly injected; the rectum is surrounded, three-fourths of an inch above the anus, by what appears to the naked eye to be a fibrous deposit; it is one-half inch in length, and had been perforated by the trocar, leaving its walls one-fourth of an inch thick; it is quite firm under pressure, and cuts with a very perceptible grating noise; the rectum, immediately above this, was four or five times its normal size. There was no microscopic examination made of the part. The bladder, kidneys, and other abdominal viscera were perfectly healthy. No other cavity was examined.

*Remarks.*—Death in such cases is the rule; my case, as the record shows, forms no exception to it, and presents in this respect nothing peculiar. When the case was examined before the operation, it exhibited all the appearances of the most simple form of imperforate rectum, viz.: a simple membrane extending across the bowel, with these exceptions, that there was no convexity, or pouting of the membrane downward, and very slight pressure against the probe when the child cried. The absence of these symptoms is readily accounted for, by the dense deposit found surrounding the rectum, in a ferule-like manner, or rather taking the place of the rectum. The rectum above was continuous with the deposit, and its large size here may have been the result of the accumulation of meconium and gas after the birth of the child. In the *Boston Medical and Surgical Journal* for February, 1858, p. 512, I notice that Dr. J. B. S. Jackson thinks that the enlargement in the lower extremity of the bowel, in imperforate anus, is not due entirely to an accumulation after birth.

In all of the recently reported cases that I have examined, and in several surgical works, I can find nothing spoken of that resembles the one above-reported, in regard to the fibrous deposit; but in the *Medical Facts and Observations*, London, 1791, vol. i. p. 238, I find a case reported by "Mr. Edward Ford, Surgeon of the Westminster General Dispensary," accompanied by a drawing of the rectum and deposit, as found at the post-mortem examination. This drawing is a true representation of the parts, as found in the case above. He says, in speaking of his "dissection," that "the rectum terminated in a blind pouch, at the distance of an inch from the anus, in the hollow of the sacrum. The space between the intestine and anus was lined by an inelastic ligamentous substance, which would probably have produced much inconvenience to the patient in retaining his stools, had the operation performed protracted his existence."

He does not mention whether there was or was not the grating noise under the knife; otherwise, so far as I can see, the two cases are precisely alike pathologically; and had my patient lived, I can readily understand that he would have presented all the symptoms of one laboring under well-marked stricture of the rectum.

DR. HARRIS related a case of a child which had been operated on for imperforate anus forty-eight hours after birth. The intestine above the stricture formed a *cul de sac*, which was very large. The child lived twenty-two months after the operation, but was never in very good health. It died of a diarrhœa, brought on by eating fruit. Seven cherry-stones were found above the seat of stricture; they could not pass through the opening.

DR. GROSS thought that in very many cases there is a dense, fibro-cellular band in which the bowels terminate. It forms a barrier, an inch to an inch and a half in length, and gives rise to much difficulty, even after the operation.

DR. HEWSON believed that most cases of imperforate anus arise from arrested development, and not from morbid growths. In the fœtus these parts are all developed from the same structure, and in the earliest stage of fœtal life there is no communication between the anal orifice and the bowel. Now, in some cases of imperforate rectum, we meet with an opening into the vagina, which, as the rectum and vagina in fœtal life communicate, would prove many of the cases to depend upon an arrest of development.

DR. STILLÉ did not think that this could here be the case, since under those circumstances no increase of fibrous tissue occurred. Nor did he believe that the case was one where the bowel terminated in a fibro-cellular band. The fibrous substance here existed not only between the two parts of this bowel, but in large quantities around the termination of the upper portion of the rectum. The great thickness of the fibrous formation prevented this portion from being pressed down in a sac-like form.

*Serous Apoplexy of the Retina.*—DR. ADDINELL HEWSON related the history of the case of an Irishman, a bootfitter by trade, affected with this lesion. The patient was aged thirty-seven years, married; of a scrofulous diathesis; partially bald; and had grayish-blue eyes. He was an industrious man, and followed his trade steadily from 6 A.M. to 12 P.M., and was obliged to tax his sight at his work. But, although he was not intemperate, he was in the frequent habit of dram-drinking.

One day in the month of August last he went out through the broiling sun, about noon, into the yard to urinate. On his return to the house he was suddenly seized with a peculiar dimness and imperfection of vision in his left eye. One-half of everything he looked at with this eye appeared to possess a bluish-yellow or greenish hue, and to be hollow or flat as the object was either really plain or convex. Dr. Hewson saw him six weeks after this occurrence, these symptoms were still present though not as intense as they were at first. There was to the unaided vision nothing apparently the matter with the eye. The pupil was natural, regular, uniform, and responded promptly to the light. The ball seemed very firm. The ophthalmoscope revealed an effusion between the hyaloid and retina, of a reddish-yellow color, and extending over the right hemisphere of the eye. Through this effusion the vessels of the retina could be but indistinctly seen; they appeared large and seemed to project forward, as though the effusion had taken place behind as well as in front of the retina. This fact could be especially well perceived at the points where the vessels passed from the circumference of the optic nerve to be distributed to the retina. This patient was blistered very frequently on the temple, and put on Plummer's pill, gr. iij., thrice daily. Under this treatment he rapidly recovered his vision and was quite well by the end of October. During the progress of his case he was frequently examined, and the absorption of the effusion readily noted.

*Cancer of the Liver and Mesenteric Glands.*—These specimens, which had been previously exhibited to the Society, were removed by DR. BRAGG from a patient under the care of Dr. S. W. MITCHELL at St. Joseph's Hospital. The following description was given :—

The liver and mesenteric glands were markedly cancerous. The liver was also in a state of fatty alteration and greatly enlarged, so that the stomach was displaced and thrust to the left side,—the cardiac portion being in the left hypochondriac region, and the pyloric extremity in the left iliac fossa. The organ was enormously distended, and presented numerous ulcerations and two small cicatrices upon its mucous surface. The cancerous tissue was soft, and composed principally of large cells and free nuclei. The cancerous portions of the liver were light in color, and level with the surface of the organ. All other organs were healthy.

The more remarkable points in connection with this case were the total absence of pain and of burning, even when irritating condiments were used as food. Pressure over the region of the stomach also gave no pain, but pressure below the ensiform cartilage really affected the liver and not the stomach, which was, as above stated, completely dislocated. Vomiting was almost the only evidence of the presence of ulcers in the stomach, and this symptom easily yielded to $\frac{1}{3}$ gr. doses of nitrate of silver. The existence of cancer of the liver was diagnosticated during life ; but the great bulk of what was felt as a solid tumor in the epigastric region was due to the increase of the size of the liver from fatty deposit, while the mass of the cancer lay behind both liver and stomach in the mesenteric glands.

